# Study protocol – Interventions to reduce musculoskeletal occupational injury in surgeons and interventionalists: A systematic review

**DOI:** 10.1016/j.isjp.2019.04.002

**Published:** 2019-04-18

**Authors:** Kiron Koshy, Habib Syed, Andrzej Luckiewicz, Miss Lorraine Harry

**Affiliations:** a Royal Victoria Infirmary, Newcastle upon Tyne Hospital Foundation Trust, Newcastle upon Tyne, UK; b Brighton and Sussex Medical School, Brighton, UK; c University College London Medical School, London, UK; d Queen Victoria Hospital NHS Foundation Trust, East Grinstead, UK

**Keywords:** Ergonomics, Occupational injury, Surgical ergonomics, Operating theatre, Musculoskeletal injury

## Abstract

Musculoskeletal occupational injury is prevalent within the surgical community. This is a multi-factorial issue, but is contributed to by physical posture, environmental hazards and administrative deficiency. There is growing awareness of this issue, with several behavioural, educational and administrative techniques being employed. The literature on this topic is, however, sporadic and difficult to access by healthcare practitioners.

The primary aim of this systematic review is to evaluate the literature on the current interventions used to minimise musculoskeletal occupational injury in surgeons. This review will focus on administrative, human factor interventions and ergonomics training.

## Highlights

• Musculoskeletal occupational injury is common in surgeons and is well reported in the literature.
• No reviews currently exist on interventions used to reduce musculoskeletal injury in surgeons and interventionalists.
• In this study, we aim to carry out a systematic review to evaluate the current interventions used to minimise musculoskeletal occupational injury in surgeons and interventionalists.

## 1 Review question

The primary aim of this systematic review is to evaluate the literature on the current interventions used to minimise musculoskeletal occupational injury in surgeons. This review will focus on administrative/human factor interventions and ergonomics training.

## 2 Searches

Electronic databases were searched between September 2017 – December 2018. The databases searched were EMBASE, MEDLINE, CINAHL, Google scholar, Cochrane library and NICE database.

## 3 Types of study to be included

All original studies including randomised controlled trials (RCTs), cohort studies and case series with or without matched/paired controls with n > 10. Studies were included that were published in the prior 10 years before the end of the search period. No language restriction will be applied.

## 4 Condition or domain being studied

Occupational or workplace injury has been recognised in the office environment for decades. However, this phenomenon is under-appreciated in the healthcare field. As an altruistic profession, healthcare workers often prioritise patient care over their own physical health.

Whilst a hazard in all disciplines of healthcare, some occupations are at particularly high risk [1]. It should come as no surprise that surgeons lie in this group, due in part to long periods of standing, bending and grasping in awkward positions. Other risk factors include equipment usage. Plastic surgeons often wear loupes which increase cervical loading by 40% [2], whilst other interventionalists regularly wear lead aprons which increase strain [3], [4]. A 15-pound lead apron can put approximately 300 pounds per square inch of initial pressure on the intervertebral disc [5], [6], [7], [8]. Specific procedures also appear to carry greater risk of musculoskeletal injury. Minimally invasive surgery in particular has been reported as carrying greater risk of musculoskeletal injury [1], [2], [3], [4], [5], [6], [7], [8], [9], [10], [11], [12], [13], [14], [15], [16], [17], [18], [19].

In the last decade, this issue has been increasingly recognised. A recent meta-analysis found that 68% of surgeons reported generalised pain. The most common anatomic sites affected were the back (50%), neck (48%) and arm or shoulder (43%). Fatigue, stiffness and numbness were also prevalent symptoms. Diagnoses of disc prolapse have also been found to be as high as 15% in study populations. Operating exacerbated pain in 61% of surgeons, but only 29% sought treatment for their symptoms. However, studies have reported as many as 31% of arthroplastic surgeons required surgery for their musculoskeletal injuries. Importantly, one survey demonstrated that of surgeons with past musculoskeletal complaints, 26.7 % required work leave and 40.0 % made intraoperative adjustments. This issue, therefore, has significant financial and workforce implications.

There are also psychological ramifications. A survey found that 47% of surgeons were concerned that these conditions will shorten their career. This fear is not unfounded, however, as a survey of ophthalmic plastic surgeons reported that 9.2% had stopped operating due to pain or spinal injury.

Poor health in surgeons, undoubtedly affects patient care. 30% of surgeons said that they took their own physical symptoms into account when recommending a surgical approach for their patients [20].

Different strategies to tackle these issues exist in ergonomic theory. These can be split into three categories: engineering controls; administrative controls and personal protective equipment. There are several studies in the literature describing the use of these methods in healthcare, but proposals are sporadic with no consensus on efficient and effective management.

In this systematic review we aim to summarise the literature on the current interventions used to minimise musculoskeletal occupational injury in surgeons and interventionalists, with a focus on administrative/human factor interventions.

## 5 Inclusion/exclusion criteria

Inclusion criteria:

• Peri-operative ergonomic interventions in operating theatre
• Administrative interventions to reduce musculoskeletal occupational injury

Exclusion criteria:

• Operative work taking place outside of hospital operating theatre
• Studies reporting on non-medical staff
• Studies reporting on the use of specialised equipment

## 6 Intervention(s)/exposure(s)

Administrative controls are workforce or human changes. These include taking intra-operative breaks and investigating the utility of ergonomics training.

## 7 Comparator(s)/control

Controls will be described as expressed within individual studies. This may include no intervention or a placebo.

## 8 Outcome(s)

Primary outcome measure:

• Incidence and prevalence of musculoskeletal disease

Secondary outcomes measures:

• Satisfaction score
• Operative time
• Muscle workload
• Number of errors
• Difficulty of task

## 9 Data extraction (selection and coding)

Identified studies were listed in a Microsoft Excel® 2018 database and Zotero referencing software and duplicates were excluded. The titles and abstracts were screened for relevance by the searcher with irrelevant results being discarded. Designated authors will extract the data by developing a database with standardised extraction fields where data can be inputted from each study sequentially.

The following data will be extracted from articles and populated into a Microsoft Excel® 2018 database (Microsoft, Redmond, WA, USA):

1. Author names and Year of publication
2. Number of participants and location of study
3. Participant demographics – Age range and previous injuries
4. Study design and level of evidence according to the Oxford Centre for Evidence-based Medicine.
5. Type of procedure/speciality
6. Type of intervention used
7. Number of tasks fulfilled
8. Mean follow-up length
9. Primary and Secondary outcomes.

## 10 Risk of bias (quality) statement

Risk of bias was not formally assessed.

## 11 Strategy for data synthesis

Outcomes of interest will be tabulated and displayed in descriptive or numeric form as appropriate. A meta-analysis will only be conducted if data and outcome measures retrieved are suitably homogenous.

## 12 Analysis of Sub-groups or subsets

None planned.

## 13 Dissemination of results

This systematic review will identify and evaluate the different ergonomic interventions used by surgeons and interventionalists to minimise the risk of musculoskeletal occupational injury. Consequently, this may influence the training of surgeons and affect their intra-operative practice. Based on the results of this systematic review, independent analysis and recommendations will be made to clinicians, researchers, theatre design teams and policy makers. It will be published in the English language in a peer-reviewed journal and the authors will endeavour to respond to any commentary generated. It will also be presented at national and international conferences in the fields of surgical practice and ergonomics. It will be disseminated electronically and in print to leading researchers in the field. Brief reports of the review findings will be disseminated directly to the appropriate audiences and societies via email and other modes of communication. Updates of the review could be conducted to inform and guide healthcare practice and policy should the need arise.

## Ethical approval

Nil.

## Funding

Nil.

## Author contribution

Koshy K – Study design, data collection, data analysis, writing. Syed H – Data collection, data analysis, writing. Luckiewicz A – Data collection, data analysis, writing. Harry L – Study design, writing.

## Conflicts of interest

Nil.

## Guarantor

Koshy K.

## Research registration unique identifying number (UIN)

Prospero 118250.
