# Supplementary material for: Study protocol – Interventions to reduce musculoskeletal occupational injury in surgeons and interventionalists: A systematic review
Source: Int J Surg Protoc. 2019 Apr 18;15:5–7. doi: 10.1016/j.isjp.2019.04.002 (PMC6913547; doi:10.1016/j.isjp.2019.04.002)
Supplement: Supplementary data 1 [file mmc1.pdf]

Protocol for non-randomised studies – based on the STROBE Statement
(http://www.journal-surgery.net/article/S1743-9191(14)00212-X/fulltext)

	Item No	Recommendation	Page number on which item reported
**Title and abstract**	1	(*a*) Indicate the study's design with a commonly used term in the title or the abstract	1-5
		(*b*) Provide in the abstract an informative and balanced summary of what was done and what was found	1
**Introduction**			
Background/rationale	2	Explain the scientific background and rationale for the investigation being reported	2
Objectives	3	State specific objectives, including any prespecified hypotheses	1
**Methods**			
Study design	4	Present key elements of study design early in the paper	3
Setting	5	Describe the setting, locations, and relevant dates, including periods of recruitment, exposure, follow-up, and data collection	3
Participants	6	(*a*) *Cohort study*—Give the eligibility criteria, and the sources and methods of selection of participants. Describe methods of follow-up<br>*Case-control study*—Give the eligibility criteria, and the sources and methods of case ascertainment and control selection. Give the rationale for the choice of cases and controls<br>*Cross-sectional study*—Give the eligibility criteria, and the sources and methods of selection of participants	n/a
		(*b*) *Cohort study*—For matched studies, give matching criteria and number of exposed and unexposed<br>*Case-control study*—For matched studies, give matching criteria and the number of controls per case	n/a
Intervention	7	Description of the intervention, pre-op work up, operative details, post-op regime, etc	3
Variables	8	Clearly define all outcomes, exposures, predictors, potential confounders, and effect modifiers. Give diagnostic criteria, if applicable	3
Data sources/ measurement	9	For each variable of interest, give sources of data and details of methods of assessment (measurement). Describe comparability of assessment methods if there is more than one group	1-3
Bias	10	Describe any efforts to address potential sources of bias	5
Study size	11	Explain how the study size was arrived at	1-3
Quantitative variables	12	Explain how quantitative variables were handled in the analyses. If applicable, describe which groupings were chosen and why	3
Statistical methods	13	(*a*) Describe all statistical methods, including those used to control for confounding	4
		(*b*) Describe any methods used to examine subgroups and interactions	3
		(*c*) Explain how missing data were addressed	2-4
		(*d*) *Cohort study*—If applicable, explain how loss to follow-up was addressed<br>*Case-control study*—If applicable, explain how matching of cases and controls was addressed<br>*Cross-sectional study*—If applicable, describe analytical methods taking account of sampling strategy	n/a
		(*e*) Describe any sensitivity analyses	3
